# Mediterranean Lifestyle in Relation to Cognitive Health: Results from the HELIAD Study

**DOI:** 10.3390/nu10101557

**Published:** 2018-10-20

**Authors:** Costas A. Anastasiou, Mary Yannakoulia, Meropi D. Kontogianni, Mary H. Kosmidis, Eirini Mamalaki, Efthimios Dardiotis, Giorgos Hadjigeorgiou, Paraskevi Sakka, Angeliki Tsapanou, Anastasia Lykou, Nikolaos Scarmeas

**Affiliations:** 1 Department of Neurology, Aiginition Hospital, National and Kapodistrian University of Athens Medical School, 11528 Athens, Greece; acostas@hua.gr (C.A.A.); tsapanou@hotmail.com (A.T.); 2 Department of Nutrition and Dietetics, Harokopio University, 17671 Athens, Greece; myianna@hua.gr (M.Y.); meropikont@gmail.com (M.D.K.); eir.mamalaki@gmail.com (E.M.); 3 Laboratory of Cognitive Neuroscience, School of Psychology, Aristotle University of Thessaloniki, 54124 Thessaloniki, Greece; kosmidis@psy.auth.gr; 4 Department of Neurology, Faculty of Medicine, University of Thessaly, 41500 Larissa, Greece; ebsdar@gmail.com (E.D.); gmhadji@yahoo.com (G.H.); 5 Department of Neurology, Medical School, University of Cyprus, 2408 Nicosia, Cyprus; 6 Athens Association of Alzheimer’s Disease and Related Disorders, 11636 Marousi, Greece; vsakka@ath.forthnet.gr; 7 Taub Institute for Research in Alzheimer’s Disease and the Aging Brain, The Gertrude H. Sergievsky Center, Department of Neurology, Columbia University, New York, NY 10032, USA; 8 Independent Researcher, 34100 Chalkida, Greece; alykou@googlemail.com

**Keywords:** brain health, cognition, dietary patterns, instrumental activities of daily living, lifestyle, nutrition

## Abstract

Many lifestyle factors have been linked to cognitive function but little is known about their combined effect. An overall lifestyle pattern for people living in the Mediterranean basin has been proposed, including diet, but also physical activity, sleep and daily living activities with social/intellectual aspects. We aimed to examine the associations between a combination of these lifestyle factors and detailed cognitive performance. A total of 1716 participants from the Hellenic Longitudinal Investigation of Ageing and Diet (HELIAD), a population-based study of participants ≥65 years, were included in this analysis. Lifestyle factors were evaluated using standard, validated questionnaires and a Total Lifestyle Index (TLI) was constructed. Cognitive outcomes included mild cognitive impairment (MCI) diagnosis, a composite z-score (either continuous or with a threshold at the 25th percentile) and z-scores for five cognitive domains. A higher TLI was associated with 65% reduced odds for MCI in the non-demented individuals and 43% reduced odds for low global cognition when MCI participants were excluded, a risk reduction equivalent to 9 and 2.7 fewer years of ageing, respectively. Each lifestyle factor was differentially associated with domain-specific cognitive performance. Our results suggest that a TLI, more so than single lifestyle parameters, may be related to cognitive performance.

## 1. Introduction

Population ageing is poised to become one of the most significant social transformations of the twenty-first century. The number of people aged 60 years or above is expected to more than double by 2050 and more than triple by 2100 [1]. This shift has produced new challenges in healthcare systems, with neuro-degenerative diseases emerging as a prevalent medical condition in the elderly population. It has been estimated that 35.6 million people lived with dementia worldwide in 2010, with numbers expected to almost double every 20 years [2]. Dementia is a condition that is mostly driven by increased life expectancy, as age-specific incidence of dementia seems to be constant or even decline in high-income countries [3]. Even if age-related cognitive decline does not progress to dementia, non-clinical cognitive impairment has been associated with increased mortality rates, as well as with other conditions relevant to the older population, such as the risk of falls and serious injury [4] or with a decreased quality of life [5]. Thus, slowing down the cognitive decline related to ageing or even prevention of its clinical manifestations has emerged as a public health priority.

A vast literature supports a complex gene–environment interaction leading to Alzheimer’s disease and other dementias [6]. Modifiable lifestyle factors are an important part of such potential environmental influences that may modify the inherent genetic risk for cognitive impairment and dementia. A recent analysis of population-based data suggests that one third of Alzheimer’s disease cases, the most prevalent type of dementia, might be attributable to potentially modifiable risk factors [7]. Nutrition is a modifiable lifestyle factor and an increasing body of evidence suggests a link between nutrition and brain health. Among several dietary patterns, the Mediterranean dietary pattern (MeDi) is the most extensively studied and at least three meta-analyses have been published, suggesting a potentially beneficial association of high adherence to MeDi in reducing the risk of developing mild cognitive impairment, dementia or Alzheimer’s disease [8,9], and neurodegenerative diseases overall [10]. Recently, it has been proposed that the beneficial effect of MeDi may extend beyond diet into a lifestyle pattern that is characterized not only by high consumption of the core foods of people living in the Mediterranean basin, but also other lifestyle parameters, namely participation in leisure activities, including physical ones, social interaction and adequate sleep [11,12].

These additional aspects of lifestyle have been independently linked to cognitive outcomes. High or even low-to-moderate levels of physical activity have been found to be protective against age-related cognitive decline [13], and have been associated with a decreased risk of cognitive impairment or dementia incidence [14]. Notably, a systematic review of lifestyle factors related to dementia has provided a broad consensus of the available data on the protective role of leisure activities that were not only physically stimulating, but also had social and cognitive dimensions [15]. In line with this observation, a combination of activities of daily living that include social engagement, and more complex activities that include some cognitive capabilities, have been associated with a decreased risk of dementia [16], or have even proved to be sensitive to cognitive differences within the normal spectrum [17]. Sleep parameters are also under study in relation to cognition. Low sleep quality has been linked with incident cognitive impairment [18], and sleep inadequacy and daytime sleepiness have been identified as significant risk factors for dementia [19].

There is relatively limited information regarding the combined association of the aforementioned lifestyle parameters with cognitive function. High adherence to MeDi and high levels of physical activity have been independently associated with a decreased risk of Alzheimer’s disease, with their combination being more potent on decreasing the disease risk [20]. Another study examined six lifestyle factors (diet, exercise, church attendance, social interaction, alcohol intake and smoking) in older individuals. A pattern that was characterized by favorable scores in all parameters was associated with the lowest risk of developing Alzheimer’s disease over six years [21]. In a more recent study that evaluated nine lifestyle factors, five of them were found to be protective against cognitive decline; these were vegetable and fish consumption, regular exercise, smoking and light-to-moderate alcohol consumption. Those who had beneficial levels in at least three of the five, but also low income, had slower cognitive decline over two years [22]. Overall, the available data on the combined influence of multiple lifestyle factors in cognitive health are inadequate, either limited by the number of lifestyle factors examined in combination or by the non-comprehensive evaluation of these factors.

The aim of this work was to evaluate the combined influence of lifestyle factors that have been incorporated in the recently developed Mediterranean lifestyle pattern (diet, physical activity, sleep, activities of daily living) [12] on cognitive performance in older adults. We examined associations with both global cognition as well as domain-specific cognitive function. Following this, we combined these lifestyle factors into a single lifestyle index and examined potential associations between the lifestyle index and the cognitive parameters.

## 2. Materials and Methods

### 2.1. Setting and Study Population

Elderly (≥65 years old) men and women from the Hellenic Longitudinal Investigation of Ageing and Diet (HELIAD) were included in the present analysis. HELIAD is a large-scale, multidisciplinary investigation involving the evaluation of a substantial number of factors relevant to dementia in two cities in Greece, Larissa and Marousi. Participants were selected through random sampling from municipality records and written informed consent was provided before entering the study. The study protocol was approved by the University of Thessaly and the National and Kapodistrian University of Athens Ethics Committees. The study ascertained exhaustive information pertaining to several domains: demographics; medical history; neurological, psychiatric, and neuropsychological assessment; anthropometry; and lifestyle parameters including nutrition, physical activity, sleep and social life. Qualified neurologists, neuropsychologists and dieticians (all of them adequately trained) administered the questionnaires and conducted face-to-face interviews. Details on the scope of the study, population, design, recruitment procedures and participation rates have been presented elsewhere [23,24,25]. Below we provide some additional details on selected aspects of the evaluation that pertain to the particular analyses of the present paper.

### 2.2. Neuropshychological Evaluation and Clinical Diagnoses

Participants received a comprehensive neuropsychological assessment including all major cognitive domains: orientation (Mini Mental State Exam [26]); verbal and non-verbal memory (Greek Verbal Learning Test [27], including five learning trials of a 16-item shopping list of semantically related items, the Medical College of Georgia (MCG) Complex Figure Test—immediate and delayed recall of an abstract line drawing [28]); language (semantic and phonological verbal fluency, with the categories used being objects and the letter A [29], subtests of the Greek version of the Boston Diagnostic Aphasia Examination short form, namely, the Boston Naming Test short form, and selected items from the Complex Ideational Material Subtest, to assess verbal comprehension and repetition of words and phrases [30]); visuoperceptual ability, using every third item from the abbreviated form of Benton’s Judgment of Line Orientation [31,32] and the MCG Complex Figure Test copy condition Clock Drawing Test [33]); attention and information processing speed (Trail Making Test Part A [34]); executive function (Trail Making Test Part B, verbal fluency, anomalous sentence repetition created for the present investigation, graphical sequence test, motor programming [28], with the last two based on Luria’s method, months forwards and backwards); and a gross estimate of intellectual level using a Greek multiple choice vocabulary test [35].

The scores of each cognitive test were converted into z-scores using mean and standard deviation values of the non-demented participants. Subsequently, z-scores of individual neuropsychological tests were averaged to produce domain z-scores in memory, language, attention-speed of information processing, and executive and visual-spatial functioning. Grouping of neuropsychological tests was based on a priori neuropsychological knowledge of particular cognitive functions that each test reflects. Furthermore, domain z-scores were averaged in order to calculate a composite z-score indicating global cognitive functioning.

Diagnoses of dementia and mild cognitive impairment (MCI) were reached through diagnostic consensus meetings of all the researchers and main investigators, both neurologists and neuropsychologists, involved in the project, and were set according to international criteria (Diagnostic and Statistical Manual for Mental Disorders, DSM-IV, National Institute of Neurological and Communicative Disorders and Stroke and Alzheimer’s Disease and Related Disorders Association criteria and International Working Group on MCΙ) [36].

### 2.3. Dietary Intake and Adherence to the Meditteranean Diet

Dietary intake was evaluated with a semi-quantitative food frequency questionnaire that has been validated for the Greek population [37]. Responses were converted to daily or weekly intakes of specific food items and were grouped into food groups featuring the core foods of the traditional Greek diet [38]. Adherence to the Mediterranean pattern was evaluated through the Mediterranean Dietary Score (MedDiet Score) proposed by Panagiotakos et al. [39]. The scoring is based on the weekly consumption of 11 food groups and an individual score for each component is calculated, ranging from 0–5. For the consumption of items that are presumed to closely characterize the Mediterranean pattern (i.e., non-refined cereals, fruits, vegetables, legumes, potatoes, fish and olive oil), individuals who reported no consumption were assigned a score of 0, and scores of 1–5 were assigned for rare to daily consumption. For the consumption of foods that are presumed to diverge from this dietary pattern (i.e., meat and meat products, poultry and full-fat dairy products), participants were assigned scores on a reverse scale. For alcohol intake, it was assumed that small amounts of consumption are beneficial, while either high or no consumption are detrimental. Thus, a score of 5 was assigned for consumption of less than 300 mL of alcohol/day but above 0 mL of alcohol/day; a score of 0 was assigned for no consumption or for consumption of 700 mL/day or more; and scores of 4–1 were assigned for consumption of 600–700, 500–600, 400–500 and 300–400 mL/day (100 mL have 12 g of ethanol concentration), respectively. The total MedDiet score ranges from 0 to 55, with higher values indicating greater adherence to the Mediterranean dietary pattern.

### 2.4. Physical Activity

Assessment of physical activity was performed using a validated brief questionnaire (Athens Physical Activity Questionnaire—APAQ) [40]. This questionnaire collects data on physical activity from the week previous to the evaluation and examines the time spent in occupational activities, activities at home and recreational activities, as well as sedentary time and sleep. For each activity a specific metabolic equivalent (MET) value is given and energy expenditure in terms of kilocalories per minute is calculated based on the subject’s body weight in kilograms divided by 60. Total energy expenditure is calculated as MET·min/day or kcal/day. For the purposes of this paper, total physical activity is expressed as total MET·min/day, excluding energy expenditure during sleep.

### 2.5. Sleep Quality

Participants were asked to complete the Sleep Scale from the Medical Outcomes Study (MOS), consisting of 12 self-reported items [41]. Sleep quality was then evaluated through the Sleep Index II [42], by summing up the following questions referring to the previous 4-week period: 1. “How long did it usually take for you to fall asleep?”, 2. “Feel that your sleep was not quiet (moving restlessly, feeling tense, speaking, etc., while sleeping)?”, 3. “Get enough sleep to feel rested upon waking in the morning?” 4. “Awaken short of breath or with a headache?” 5. “Feel drowsy or sleepy during the day?” 6. “Have trouble falling asleep?” 7. “Awaken during your sleep time and have trouble falling asleep again?” 8. “Have trouble staying awake during the day?”, and 9. “Get the amount of sleep you needed?”. Each of the questions has a possible rating of 1–6, based on the frequency of the sleep problem. Sleep Index II scores range from 1 to 54, with higher scores indicating greater sleep dysfunction. In order to be concordant with the directionality of other lifestyle indices, we present data for the sleep index in reverse order, so as to represent sleep quality, with a range of 1–54 and higher scores indicating better sleep quality.

### 2.6. Instrumental Activities of Daily Living (IADL)

We chose to use the IADL-extended (IADL-x) scale to assess functionality and capabilities relating to maintenance of self and lifestyle. This is a 9-item scale including 5 cognitive leisure activities of daily living (went to classes, community volunteer work, club or center activities, went to the movies, restaurant, sporting event, visited friends or relatives in the last month) and 4 more complex/advanced activities (had difficulties in shopping, difficulties with light housework, trouble getting around the neighborhood, needed help with medication) [17]. Responses to all items are dichotomous (yes or no) and the IADL-x score ranges from 0–9, with higher values indicating a higher functional status with regard to the aforementioned activities. It has been found that this short scale is effective in predicting dementia incidence and that it is sensitive to cognitive differences within the normal spectrum of various domains of cognitive function [16,17].

### 2.7. Total Lifestyle Index (TLI)

A TLI was calculated by taking into consideration all lifestyle factors examined, and performance in each lifestyle factor in relation to the total distribution of the factor in our sample. For each factor, a score of 0 was given to an individual when the value was in the first quartile of the distribution of each specific factor (<25th percentile) and a value of 1, 2 or 3 was given when the value was within the second (≥25th percentile and <50th percentile), third (≥50th percentile and <75th percentile) or fourth (≥75th percentile) quartile respectively. TLI is the result of the sum of all four sub-scores of the lifestyle factors examined. TLI scores range from 0–12, with higher values indicating an overall beneficial lifestyle.

### 2.8. Statistical Analyses

We initially considered all participants. As a next step, in order to reduce the possibility of misreporting, we excluded participants with dementia and examined associations between TLI or individual lifestyle factors (independent variables) and each of the cognitive domains (dependent variables) using linear regression analysis. We also evaluated the odds ratio for MCI by logistic regression analyses.

Finally, in an even more conservative approach, we excluded both participants with dementia and MCI and examined the odds ratio of low global cognitive functioning (defined as a composite z-score below the 25th percentile (i.e., within the first quartile) in association with TLI or individual lifestyle factors (as independent variables).

In all models age (years), sex (dichotomous) and education (years) were entered as potential confounders. Individual lifestyle factors and TLI were entered into the models both as continuous variables and as categorical variables (comparing the first versus other quartiles). When examining individual lifestyle factors, all these factors were entered simultaneously into the same model.

Continuous variables are presented as mean values ± 1 standard deviation and categorical ones as relative (%) frequencies. Differences among groups were tested through analysis of variance with Bonferroni correction for pairwise comparisons and Pearson’s x^2^ for continuous and categorical variables, respectively. Statistical significance was set at *p* ≤ 0.05.

## 3. Results

### 3.1. Total Sample Charachteristics

Among a total of 1716 participants, 60 were diagnosed with dementia and 206 with MCI. As expected, older age and lower education were related with poor cognitive status, as seen in Table 1. With the exception of sleep, worse lifestyle was observed in dementia compared to the normal cognition group. TLI was worse for both the dementia and the MCI groups, compared to the normal cognition group.

### 3.2. Excluding Participants with Dementia

Better TLI was associated with better global cognitive functioning, as seen in Table 2. Analysis by cognitive domain, with each domain entered into the model separately, revealed significant positive associations of TLI with memory, executive visual-spatial and language domains, but not for attention-speed of information processing.

We further examined the associations between each lifestyle factor and cognitive z-scores, as seen in Table 3. Better diet was related to better memory, visual–spatial and language function. Subjects who were more physically active had better memory, were cognitively faster and had better executive skills. Better sleep was related to better visual–spatial function. Higher functionality was associated with better language and executive function performance. All components of TLI, with the exception of sleep, were related to global cognitive performance.

The odds for MCI were significantly associated with TLI, either expressed as a continuous variable or as quartiles, as seen in Table 4. Subjects in the highest TLI quartile had 55% reduced odds for MCI. To put this into perspective, this was equivalent to a risk reduction of nine fewer years of cognitive ageing (odds ratio for age in the same model of 1.061, *p* < 0.001).

**Figure 1:**
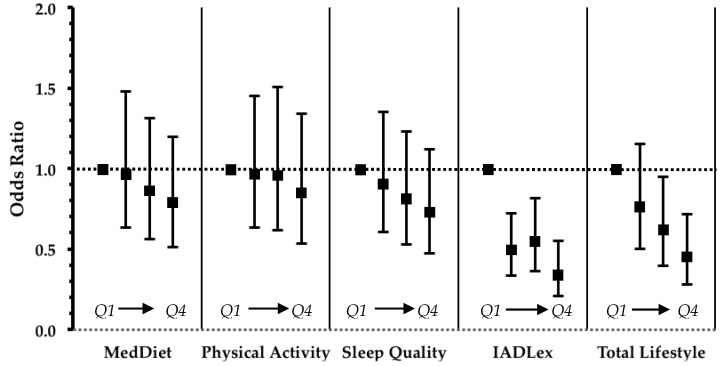
Results from logistic regression that evaluated the impact of lifestyle factors and TLI on the odds ratio of MCI in non-demented participants. Values are odds ratios with error bars representing 95% confidence intervals. In all models age, sex and education were entered as confounders. Statistically significant linear trends at *p* < 0.05 were observed for IADLex and Total Lifestyle. MedDiet: Mediterranean diet, IADLex: instrumental activities of daily living extended scale. Q: quartile (*Q1* lowest, *Q4* highest).

Odds ratios for MCI in association with individual lifestyle factors are presented in Figure 1. Of the four factors, higher functionality was significantly associated with lower MCI odds.

### 3.3. Excluding Both Participants with Dementia and MCI

Considering only individuals in the normal spectrum of cognitive function, higher levels of education and younger age were related to better cognitive performance, as seen in Table 5. More favorable individual lifestyle factors and higher values of TLI were observed with increasing quartiles of global cognitive functioning. In multivariate linear regression, adjusted for age, sex and education, TLI was associated with global cognitive functioning (beta = 0.058, *p* = 0.012).

TLI was associated with reduced odds for low cognitive performance in unadjusted models, as seen in Table 6. In adjusted models, individuals in the upper quartile of TLI had 43% decreased odds of low cognitive performance, equivalent to 2.7 fewer years of cognitive ageing (odds ratio for age in the same model of 1.161, *p* < 0.001).

**Figure 2:**
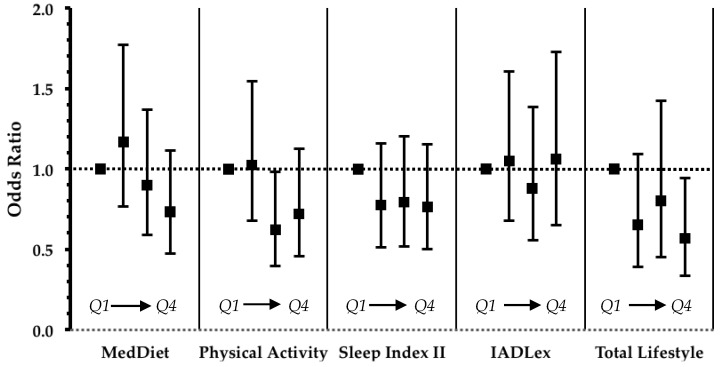
Results from logistic regression that evaluated the impact of lifestyle factors and TLI on the odds ratio of low cognitive performance in non-demented, non-MCI participants. Values are odds ratios with error bars representing 95% confidence intervals. In all models age, sex and education were entered as confounders. Statistically significant linear trend at *p* < 0.05 was observed for physical activity. MedDiet: Mediterranean diet, IADLex: instrumental activities of daily living extended scale. Q: quartile (*Q1* lowest, *Q4* highest).

Unlike TLI, in analysis of individual lifestyle factors, physical activity was the only one with a statistically significant linear trend with the odds of low cognitive performance, as seen in Figure 2. In addition, the association of the highest quartile of individual lifestyle factors with low cognitive performance did not reach statistical significance (as noted for TLI).

## 4. Discussion

Acceleration of ageing worldwide, and thus increasing incidence of neurodegenerative diseases, in combination with the fact that no efficient medical treatment for dementia has been discovered so far, have increased interest in the identification of modifiable factors that could prevent, or at least delay, the occurrence of dementia in the older population. In this paper, we provide evidence that a lifestyle index composed of four factors, namely diet, physical activity, sleep and functionality, is related to better cognitive function, raising the possibility of synergies, additive effects and/or interactions among separate lifestyle dimensions. TLI was lower in subjects with dementia and was associated with reduced odds of MCI when we excluded participants with dementia, but also with reduced odds of low cognitive performance in dementia- and MCI-free older adults. Thus, we can argue that noted associations seem less affected by either reporting bias or reverse causality.

The implementation of a TLI, and thus the consideration of a combination of lifestyle factors, has several advantages over the more simplistic single factor approach. Individual factors may have weak or even non-significant associations with health outcomes that cannot be detected in research investigations, but also in clinical practice. In a study of the influence of several health-related behaviors on overall mortality it was observed that every single lifestyle behavior reduced cumulative mortality by 0.62%, while a combination of more than four factors reduced the hazard ratio by as much as 30% [43]. Additionally, it has been observed that many health-related behaviors do not occur within individuals by chance, but rather they cluster [44] and may be interrelated. For example, a high diet quality has been associated with better sleep quality and lower risk of significant changes in sleep duration [45,46]. In this regard, the use of a TLI may even consider potential mediation effects and capture such clustering when used to predict a health-related outcome.

The associations between TLI and global cognitive performance observed in the present study may be considered as the combination of the influence of each lifestyle factor in specific domains of cognitive functioning. TLI was associated with almost all cognitive domains. However, when associations of individual lifestyle factors with individual cognitive domains were examined, it was observed that each lifestyle factor was related to specific domains. The impact of each lifestyle factor on performance in specific domains of cognitive functioning remains to a large extent an unexplored area of research.

We found a significant association of functionality with reduced MCI odds, whereas after exclusion of MCI participants and considering only subjects within the normal range of cognitive performance, we observed that physical activity was related with reduced odds for lower cognitive performance. However, both MCI and lower cognitive performance odds were lower for people in the highest quartile of the TLI, suggesting that individual lifestyle factors may also have minor, but possibly substantial, influence that can be more efficiently captured through the more holistic approach of an overall lifestyle index.

The TLI approach has emerged as an effective means for both prevention and treatment of many prevailing diseases, such as cardiovascular diseases [47]. In the area of brain health, however, fewer data have been presented so far, with studies limited either by the number of lifestyle factors assessed [20] or by the non-comprehensive evaluation of lifestyle factors into discrete domains of lifestyle (e.g., quality of overall diet, total physical activity) [21,22]. On the other hand, some clinical trials have examined such an approach. Three large-scale multi-domain interventions that incorporated changes in many lifestyle factors for the prevention of cognitive decline have been completed so far [48,49,50]. These trials included coaching for a healthier diet and increased levels of physical activity, as well as cognitive training and control of cardiovascular risk factors. The FINGER trial found a slower cognitive decline in individuals assigned to the multi-domain intervention [50], while in the other two trials (PREDIVA and MAPT trials) protective effects were observed under specific circumstances (e.g., those with more vascular factors, higher future dementia risk scores, brain amyloid positivity or carriers of the e4 allele of Apolipoprotein E) [48,49]. Although clinical trials have been widely considered as the gold standard method for the determination of causal relationships, in the case of lifestyle parameters well-designed observational studies are also of great value, as they may capture long-term exposures and they may allow exploration of the associations between many more potential lifestyle factors and health outcomes, and may also permit the investigation of the combined influence of lifestyle factors.

Certain limitations of this study deserve consideration. This is a cross-sectional approach that cannot provide causal relationships but only formulate hypotheses for future research. Although we took precautions by gradually excluding individuals with altered cognitive function and by adjusting for major variables, reverse causality and the influence of residual unmeasured confounding variables cannot be entirely excluded.

Our study has several strengths. A major advantage of the study is the fact that we constructed a lifestyle index that was based on observational data of a Mediterranean population, with core health-related behaviors, such as diet, that have been widely identified to be linked with reduced morbidity and mortality rates [9,10]. Our sample is population representative and we used validated tools for the assessment of each lifestyle factor evaluated. We also administered a comprehensive neuropsychological evaluation that included both a full neuropsychological battery and a diagnosis of cognitive impairment based on clinical consensus of an experienced team of investigators, with use of standard criteria.

## 5. Conclusions

Our data are in support of a beneficial influence of four lifestyle factors, namely diet, physical activity, sleep quality and functionality, on cognitive performance in the elderly. We constructed a lifestyle index that can capture a combination of these lifestyle factors and is associated with cognitive performance of an extended spectrum of cognitive function, even when only individuals in the normal range of cognitive functioning were included in the analysis. Thus, our results may form the basis for the maintenance of a healthy ageing brain. Future research directions may include the validation of such an index on a prospective basis, and then the development of a lifestyle index linked with a simple scoring system for use in clinical practice.

## Figures and Tables

**Table 1:** Socio-demographic characteristics and lifestyle factors of the total sample and by diagnosis.

	All	Normal Cognition	MCI	Dementia	*p* ^1^
	*n* = 1716	*n* = 1450	*n* = 206	*n* = 60	
Age, years	72.9 ± 6.1	72.4 ± 6.0	74.8 ± 5.5 *	77.8 ± 5.4 *^, #^	<0.001
Sex, % male	40.4%	39.9%	42.2%	48.3%	0.363
Education, years	7.7 ± 4.8	7.9 ± 4.7	6.9 ± 5.2 *	5.8 ± 5.0 *	<0.001
Illiteracy, % yes	5.1%	3.9%	10.2%	15.2%	<0.001
MMSE score Literate, range 0–30 Illiterate, range 0–23	26.8 ± 3.1 18.9 ± 3.7	27.4 ± 2.3 20.4 ± 2.0	24. 5± 3.3 * 18.6 ± 2.3 *	17.9 ± 6.2 *^, #^ 12.0 ± 4.2 *^, #^	<0.001 <0.001
MedDiet Score, range 0–55	33.0 ± 4.5	33.2 ± 4.5	32.6 ± 4.4	31.4 ± 4.8 *	0.003
Physical Activity, per 200 MET·min/day	7.4 ± 1.4	7.5 ± 1.4	7.3 ± 1.4	6.8 ± 1.3 *	<0.001
Sleep Quality, range 1–54	37.3 ± 7.8	37.4 ± 7.7	36.4.6 ± 7.6	36.1 ± 9.0	0.117
IADLex, range 0–9	4.5 ± 1.3	4.6 ± 1.2	4.1 ± 1.2 *	2.6 ± 1.6 *^, #^	<0.001
Total Lifestyle Index, range 0–12	6.1 ± 2.4	6.3 ± 2.3	5.4 ± 2.4 *	4.2 ± 2.3 *^, #^	<0.001

^1^*p*-values of overall effect in analysis of variance (continuous variables) or Pearson’s chi-square test (categorical variables); *, ^#^ indicate a statistically significant difference compared to normal cognition and MCI groups, respectively. Values are means ± 1 standard deviation. MCI: mild cognitive impairment, MMSE: Mini-Mental State Examination, MedDiet: Mediterranean diet, IADLex: instrumental activities of daily living extended scale.

**Table 2:** Results from linear regression analyses that evaluated the association between Total Lifestyle Index (TLI) (independent variable) and cognitive domains (dependent variables) in non-demented participants.

Cognitive Domains	Beta	*p*	R^2^
Memory	0.098	<0.001	0.301
Executive	0.071	<0.001	0.388
Visual-Spatial	0.068	0.003	0.255
Language	0.072	<0.001	0.478
Attention-Speed	0.039	0.084	0.287
Composite	0.085	<0.001	0.477

In all models age, sex and education were entered as confounders. Statistically significant results at *p* ≤ 0.005 are indicated in bold. Each cognitive domain was entered into the model separately. All regression models were significant overall at *p* < 0.001.

**Table 3:** Results from multiple linear regression analysis that evaluated the association between lifestyle factors composed TLI (independent variables) and z-scores of cognitive performance (dependent variables) in non-demented participants.

Cognitive Domains	MedDiet Score		Physical Activity		Sleep Quality		IADLex		R^2^
	Beta	*p*	Beta	*p*	Beta	*p*	Beta	*p*	
Memory	0.059	0.007	0.057	0.012	0.037	0.091	0.037	0.090	0.304
Executive	0.016	0.437	0.044	0.037	−0.002	0.931	0.085	<0.001	0.391
Visual-Spatial	0.056	0.013	0.037	0.121	0.054	0.016	−0.021	0.350	0.257
Language	0.044	0.019	0.016	0.416	0.016	0.405	0.052	0.006	0.478
Attention- Speed	−0.021	0.354	0.066	0.007	0.001	0.951	0.041	0.074	0.290
Composite	0.039	0.039	0.056	0.004	0.023	0.210	0.051	0.007	0.478

In all models age, sex and education were entered as confounders. Statistically significant results at *p* ≤ 0.05 are indicated in bold. MedDiet: Mediterranean diet, IADLex: instrumental activities of daily living extended scale.

**Table 4:** Results from logistic regression analysis that evaluated the association between TLI and MCI (dependent variable) in non-demented participants.

	Total Lifestyle Index (Continuous)		Total Lifestyle Index (in Quartiles)		
	OR [95% CI]	*p*	OR [95% CI]	*p*	*p-Trend*
Unadjusted	0.883 [0.818–0.952]	0.001	*Q1:* 1 (reference)		<0.001
			*Q2:* 0.695 [0.460–1.052]	0.085	
			*Q3:* 0.519 [0.339–0.794]	0.003	
			*Q4:* 0.342 [0.216–0.540]	<0.001	
Adjusted ^1^	0.925 [0.855–1.001]	0.054	*Q1:* 1 (reference)		0.001
			*Q2:* 0.757 [0.497–1.152]	0.193	
			*Q3:* 0.614 [0.397–0.950]	0.028	
			*Q4:* 0.446 [0.277–0.717]	0.001	

^1^ age, sex and education were entered as confounders. Statistically significant results at *p* ≤ 0.005 are indicated in bold. MCI: mild cognitive impairment, OR: odds ratio, CI: confidence interval, Q: quartile (*Q1* lowest, *Q4* highest).

**Table 5:** Socio-demographic characteristics, lifestyle factors and TLI by quartiles of composite z-score in non-demented, non-MCI participants.

	*Q1*	*Q2*	*Q3*	*Q4*	*p* ^1^
Age, years	76.4 ± 5.3	73.1 ± 5.1	71.4 ± 5.4	69.7 ± 6.2	<0.001
Sex, % male	40.4%	39.9%	42.2%	48.3%	0.363
Education, years	4.3 ± 2.9	6.0 ± 2.9	8.8 ± 4.0	12.4± 4.2	<0.001
MedDiet Score, range 0–55	32.2 ± 4.4	33.1 ± 4.4	33.3 ± 4.5	34.1 ± 4.5	<0.001
Physical Activity, per 200 MET·min/day	7.3 ± 1.4	7.5 ± 1.5	7.5 ± 1.4	7.6 ± 1.3	0.033
Sleep Quality, range 1–54	36.2 ± 8.0	36.7 ± 7.9	38.8 ± 6.9	38.0 ± 7.8	<0.001
IADLex, range 0–9	4.4 ± 1.2	4.5 ± 1.1	4.7 ± 1.2	4.9 ± 1.4	<0.001
Total Lifestyle index, range 0–12	6.2 ± 2.1	6.7 ± 2.0	7.0 ± 2.2	7.5 ± 2.1	<0.001

^1^*p*-values of overall effect in analysis of variance (continuous variables) or Pearson’s chi-square test (categorical variables). Values are means ± 1 standard deviation. Q: quartile (*Q1* lowest, *Q4* highest).

**Table 6:** Results from logistic regression analysis that evaluated the association between Total Lifestyle Index and low cognitive performance (dependent variable) in non-demented, non-MCI participants.

	Total Lifestyle Index (Continuous)		Total Lifestyle Index (in Quartiles)		
	OR [95% CI]	*p*	OR [95% CI]	*p*	*p-Trend*
Unadjusted	0.824 [0.768–0.883]	<0.001	*Q1:* 1 (reference)		<0.001
			*Q2:* 0.545 [0.358–0.829]	0.005	
			*Q3:* 0.567 [0.354–0.910]	0.019	
			*Q4:* 0.324 [0.214–0.492]	<0.001	
Adjusted ^1^	0.920 [0.845–1.003]	0.057	*Q1:* 1 (reference)		0.076
			*Q2:* 0.653 [0.389–1.095]	0.106	
			*Q3:* 0.801 [0.450–1.424]	0.450	
			*Q4:* 0.567 [0.339–0.947]	0.030	

^1^ age, sex and education were entered as confounders. Statistically significant results at *p* ≤ 0.05 are indicated in bold. MCI: mild cognitive impairment, OR: odds ratio, CI: confidence interval, Q: quartile (*Q1* lowest, *Q4* highest).
